# Clinical Considerations and Management of a Labially Impacted Maxillary Canine With Immediate Implant Placement and Alveolar Bone Reconstruction: A Case Report

**DOI:** 10.7759/cureus.71529

**Published:** 2024-10-15

**Authors:** Pankaj Dhawan, Sapna Rani, Simran Chadha

**Affiliations:** 1 Prosthodontics and Implantology, Manav Rachna Dental College, Faridabad, IND; 2 Prosthodontics, Manav Rachna Dental College, Faridabad, IND

**Keywords:** bone augmentation, canine impaction, dental implant, oral rehabilitation, prosthetic rehabilitation

## Abstract

A labially impacted maxillary canine is a relatively uncommon occurrence. In adult patients, management of impacted maxillary canines becomes necessary when the deciduous canine is lost, or if the impacted tooth becomes symptomatic. Orthodontic realignment is not always the preferred therapeutic approach due to the long treatment duration. Alternatively, prosthetic rehabilitation of the missing tooth with a dental implant can serve as a viable treatment option. This article describes a case involving a labially impacted permanent maxillary canine in a 36-year-old woman, who had a primary complaint of discoloration of the corresponding primary tooth, which was rehabilitated with an immediate implant and bone graft along with treatment of the discolored tooth. This case supports the use of a single implant for replacing an impacted tooth, eliminating the need for conventional tooth preparation in prosthetic rehabilitation or extended orthodontic intervention to reposition the impacted tooth. The treatment resulted in exceptional aesthetic outcomes. The combination of immediate implant placement with hard tissue augmentation facilitated alveolar bone reconstruction and shortened the overall treatment time.

## Introduction

Tooth impaction is characterized by the incomplete eruption of the tooth into its normal position, remaining embedded within the oral mucosa beyond its expected time of eruption [1]. Various terminologies in the literature describe tooth impaction as impacted teeth, submerged teeth, or delayed eruption. A canine is classified as impacted if the contralateral canine has erupted with fully developed roots at least six months prior, or if eruption is interrupted after root development is completed [2]. Maxillary canine impaction is common due to the prolonged developmental period, the deep anatomical location of their formation, and the complex eruption pathway [3]. The prevalence of impaction is highest for third molars (18.97%-30.80%), followed by maxillary canines occurring in about 1%-3% population [4]. In a comparison stratified by gender, it is more predominant in females as compared to males. The prevalence of maxillary impactions is reported to be in the range of 1.7%-4.7% while for mandibular ones it is said to be 0.07%- 1.36%. Additionally, impacted canines are more commonly located palatally (85%) than labially (15%) [5].

Treatment of impacted maxillary canines is unavoidable when they become symptomatic or when the primary canines are lost through extraction or exfoliation. Impacted teeth have several implications, like, they cause drifting of the adjacent teeth and reduce the intra-arch length. Additionally, canine impaction may contribute to infections and lesions with respect to adjacent teeth, further causing root resorption [6].

Radiographic imaging is essential for diagnosing impacted maxillary canines, utilizing various techniques such as cone beam computed tomography (CBCT) to provide a three-dimensional visualization of the tooth [6]. Traditional treatment options include extracting or exposing the unerupted tooth surgically, subsequently undergoing orthodontic therapy, interceptive methods, space recreation, or prosthetic replacement. An alternative treatment modality may also be considered as transalveolar surgical extraction of the impacted canine followed by immediate implant insertion [3]. High predictability has been observed with osseointegrated implants. Recent approaches to surgery have made it possible to achieve the perfect crown-implant association, making implant-supported crowns approximate adjacent natural teeth. High long-term success rates for single-tooth restorations have been reported by several authors [7]. From an aesthetic viewpoint, the replacement of a maxillary anterior single tooth is one of the most difficult cases to treat, and conservation of the surrounding tissues and reconstruction of the lost anatomy are of interest to clinical investigators [7].

Radicular cavities, non-restorable crowns, root fractures, and lack of periodontal support are the prevalent causes of tooth extraction and implant insertion. The presence of a primary canine with impaction of the permanent canine represents a less frequent clinical occurrence in adult patients [8]. In several cases, surgeons encounter challenges with immediate implant placement due to the presence of a jumping distance between the implant and neighboring socket walls and, also the bone deficiency observed in relation to the buccal cortical plate. This issue can be addressed by utilizing various grafting materials in conjunction with a barrier membrane [9]. This report presents a distinctive case involving replacing a labially impacted permanent canine, in conjunction with a discolored primary maxillary canine with an immediate implant along with a bone graft.

## Case presentation

**Figure 1:**
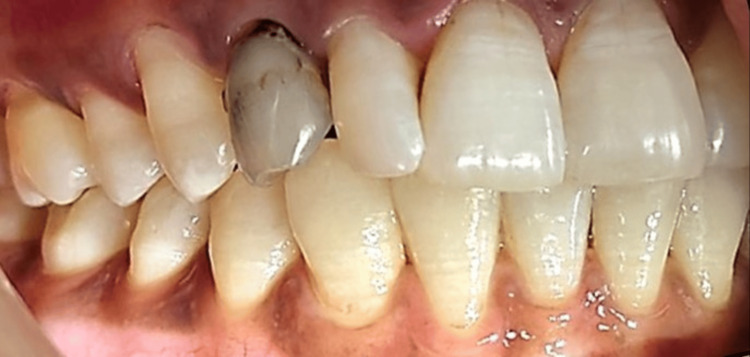
Pre-operative view showing the discolored primary canine

**Figure 2:**
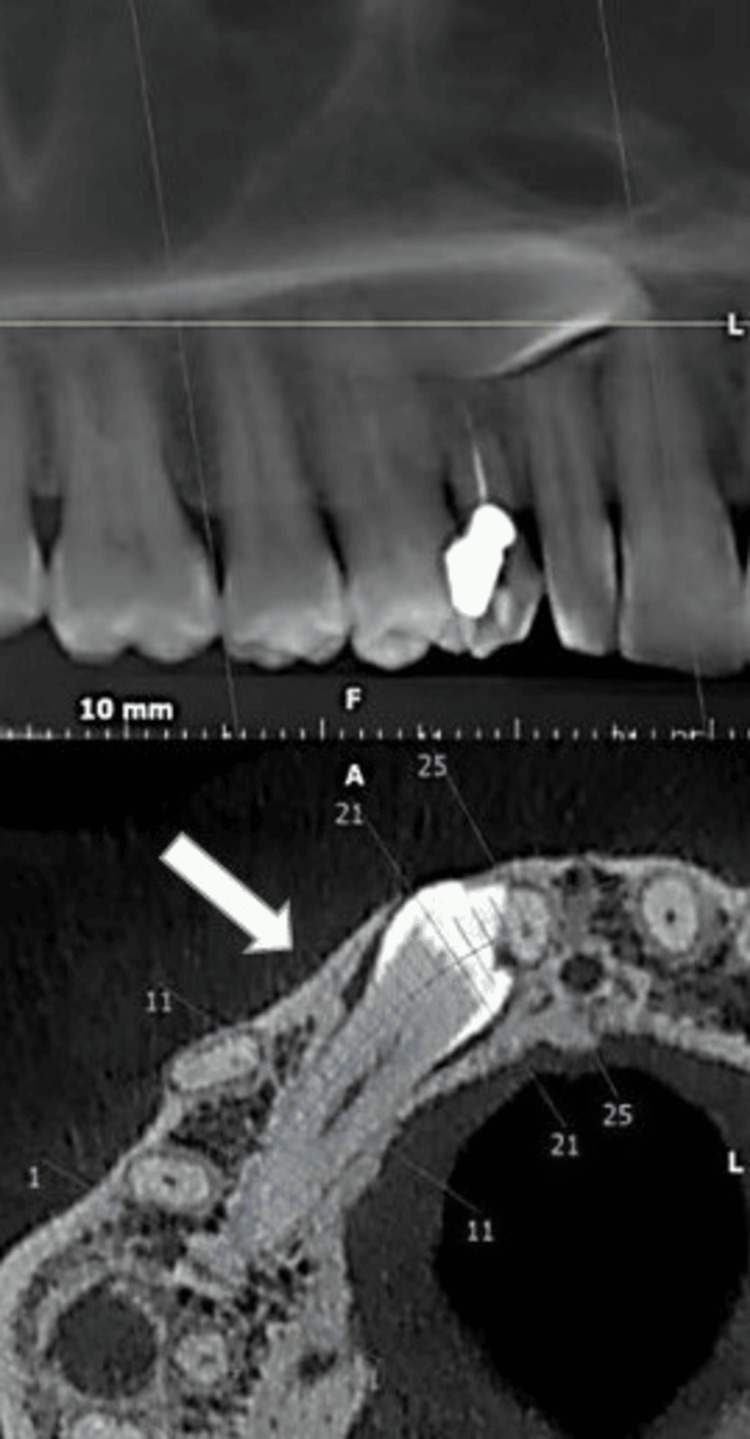
Pre-operative CBCT (cone beam computed tomography) revealing the horizontally impacted canine as indicated by the arrow

A 36-year-old female patient reported to the Department of Prosthodontics Crown and Bridge, Manav Rachna Dental College in October 2023, with the chief complaint of a discolored upper right deciduous canine (Figure 1). The clinical history of the patient was obtained. Medical history revealed the patient had hyperthyroidism disorder for few years, but it was under control after medication (levothyroxine sodium 25mg). An intraoral periapical radiograph was taken with respect to the discolored tooth, which revealed the horizontal impaction of the maxillary canine along with periapical radiolucency of the deciduous canine. Pre-operative CBCT (Figure 2) confirmed the presence of a labially impacted right maxillary canine that was near the buccal gingival margin of the right lateral incisor. The treatment option available for the patient was either extraction of the discolored primary canine and orthodontic extrusion of the permanent canine or extraction of impacted and discolored canine followed by prosthodontic management. The patient refused to undergo orthodontic treatment to bring the tooth into the arch. Consequently, transalveolar extraction of both the permanent and primary canines was opted for, followed by immediate implant placement and subsequent rehabilitation.

**Figure 3:**
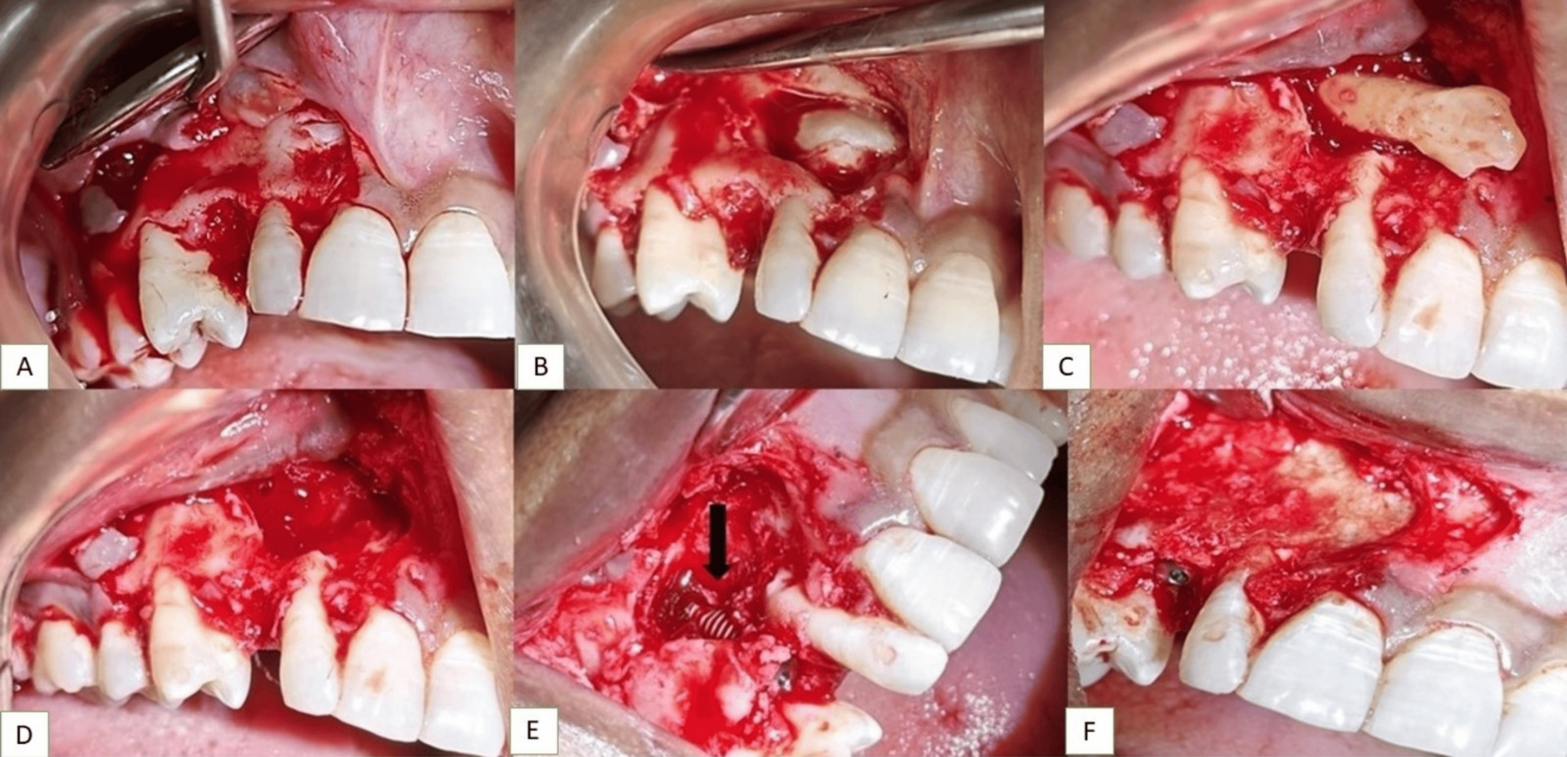
(A) Extraction of the discolored deciduous canine, (B) reflection of the full-thickness mucoperiosteal flap, (C) luxation of the impacted canine, (D) transalveolar extraction of the impacted canine, (E) immediate implant placement with respect to the impacted canine, (F) bone grafting procedure after implant placement

Written informed consent was acquired, and local anesthesia was administered via buccal and palatal injections (1% lidocaine and 1: 100,000 adrenaline). Initially, the primary discolored right maxillary canine was extracted (Figure 3A). After reflection of a 10mm full-thickness mucoperiosteal trapezoidal flap from the mesial of the right second premolar to the distal of the right central incisor using a 15c bp blade at the mucogingival junction, a buccal osteotomy was executed using a rounded carbide bur (W&H, India) under copious saline irrigation until exposure of the crown of the maxillary canine was achieved (Figure 3B). The canine was then luxated using an elevator (W&H, India) and was extracted (Figures 3C, 3D).

**Figure 4:**
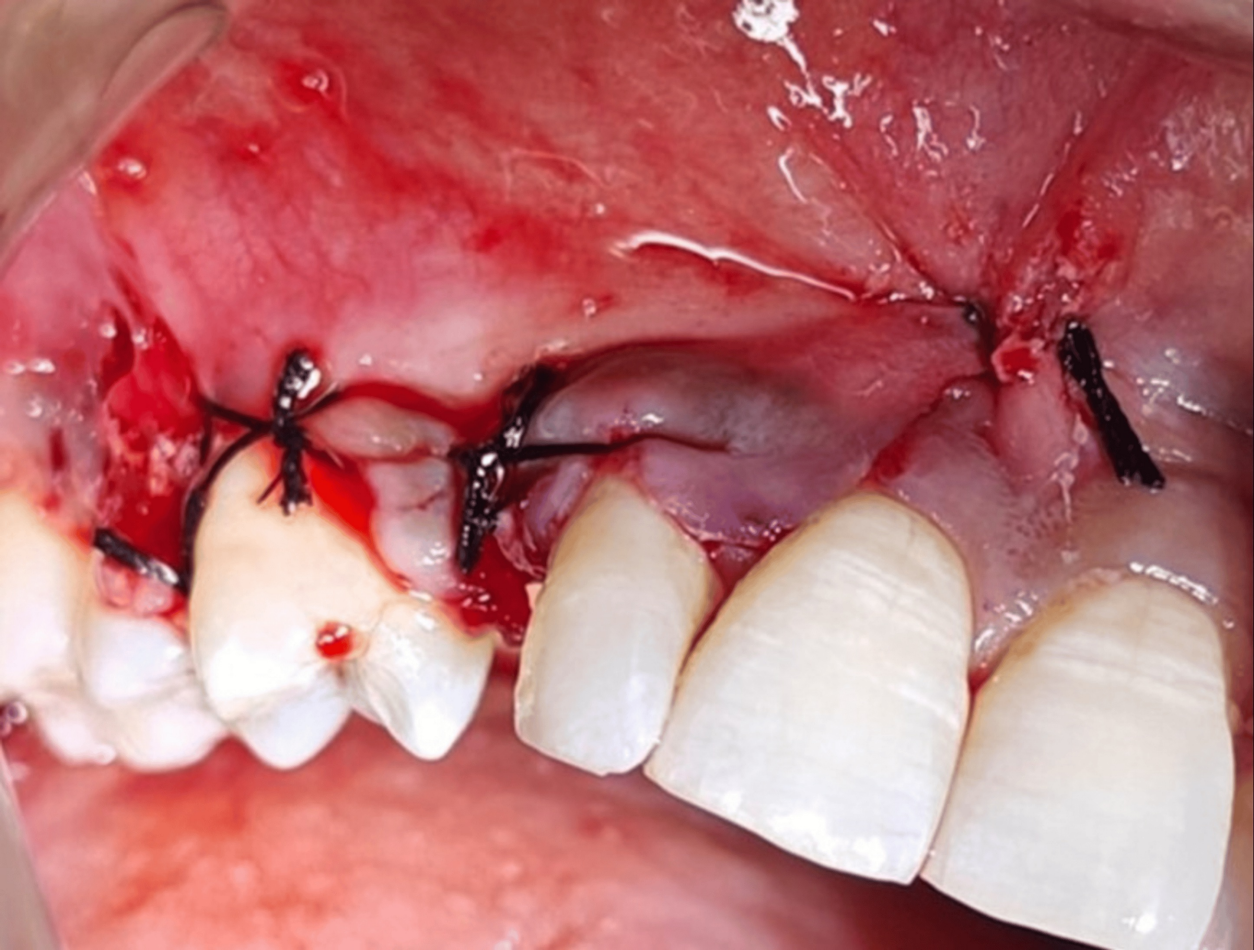
Flap closure after suturing

At this point, the exposure site was curettaged, for granulation tissue removal. A conical screw-shaped blasted implant 3.75x13mm (MIS, Dentsply), was placed, with a torque of 30 N (Figure 3E). This facilitated the mechanical assessment of bone quality and the evaluation of primary stability. A buccal bone deficiency persisted, resulting in a segment of the implant without adequate bone enclosure, so the implant and bone around it were covered with a combination of bovine bone mineral, BioOss bone graft (Geistlich Bio-Oss), autogenous graft and, subsequently layered by synthetic bone graft material made of calcium phosphosilicate (CPS) particles, polyethylene glycol, and glycerine (NovaBone dental putty) and collagen membrane (Figure 3F). The flap was sutured using half-circle braided silk 3-0 Ethicon sutures (Figure 4).

Post-operatively, the patient was administered oral antibiotics and analgesics, amoxicillin with clavulanic acid (625 mg, tab, two times a day), aceclofenac with serratiopeptidase (325 mg, tab, two times a day after food), and 0.2% chlorhexidine rinses after meal for three consecutive days. Also, the patient was suggested to have a liquid diet for a few days and maintain proper oral hygiene. The patient was reviewed after three days, and the wound was cleaned using 9% povidone iodine solution. At the time of suture removal, after one week of surgery, the soft tissues demonstrated a favorable healing response, exhibiting no signs of recession and maintaining the integrity of the interdental papillae.

**Figure 5:**
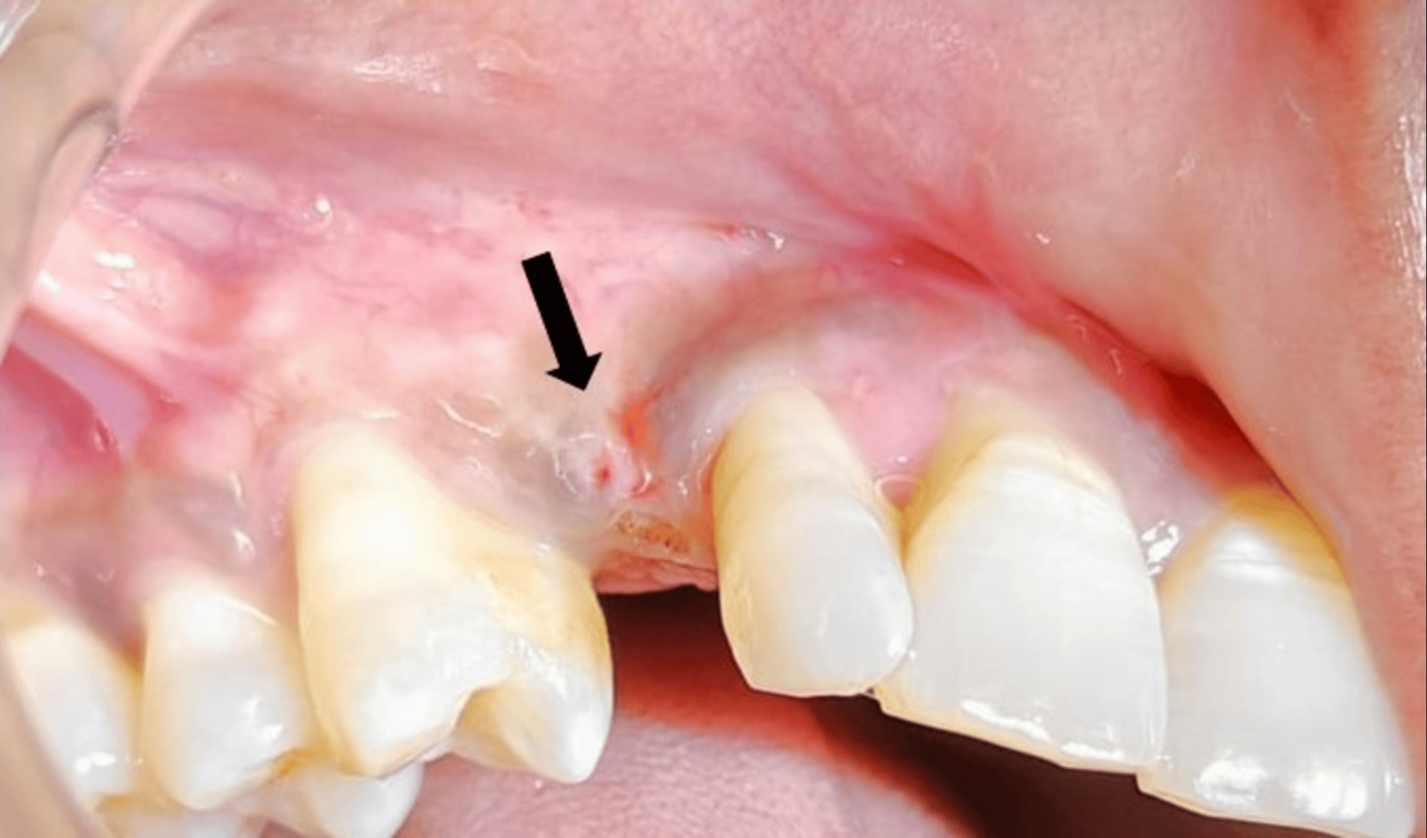
Post-operative healing after four weeks

**Figure 6:**
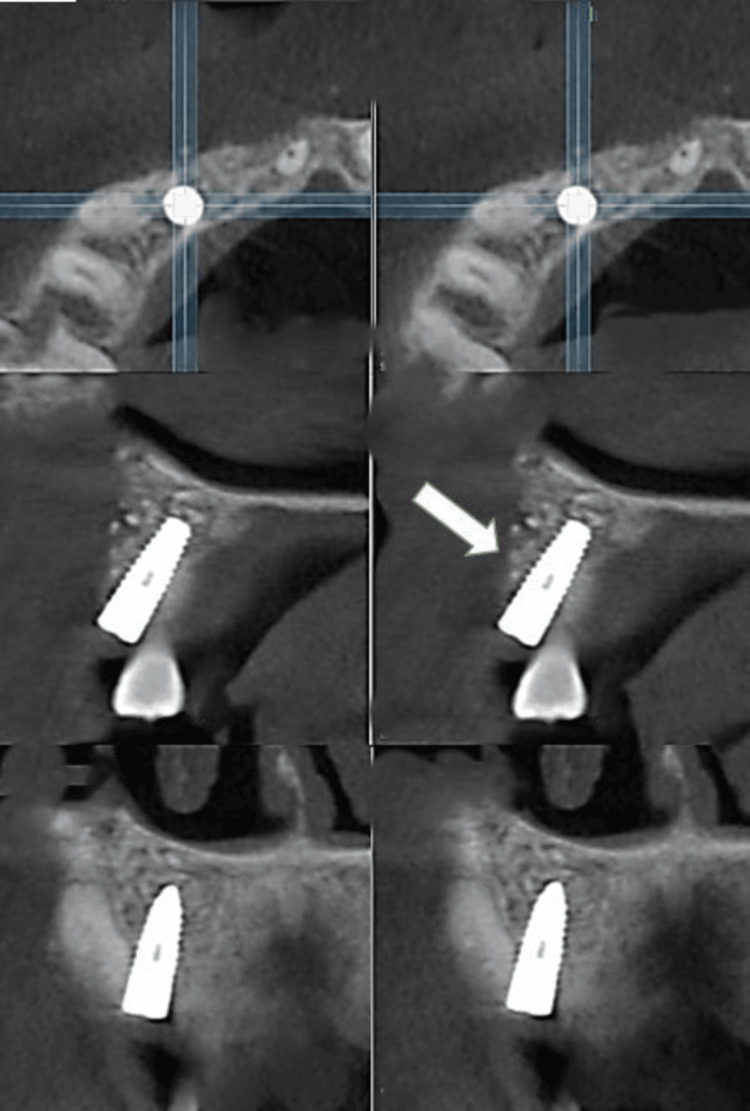
Sagittal view of the post-operative CBCT scan showing the osseointegrated implant CBCT: Cone beam computed tomography

The patient was called for follow-up after four weeks (Figure 5) and a post-operative CBCT scan was performed (Figure 6) which demonstrated bone development on the buccal aspect of the implant and satisfactory integration. The qualitative assessment of the maxilla revealed adequate graft density along the labial aspect of the implant. A partially re-mineralized socket (of previously impacted tooth 13) was seen along with endosteal new bone formation which indicated good prognosis for the treatment plan. 

**Figure 7:**
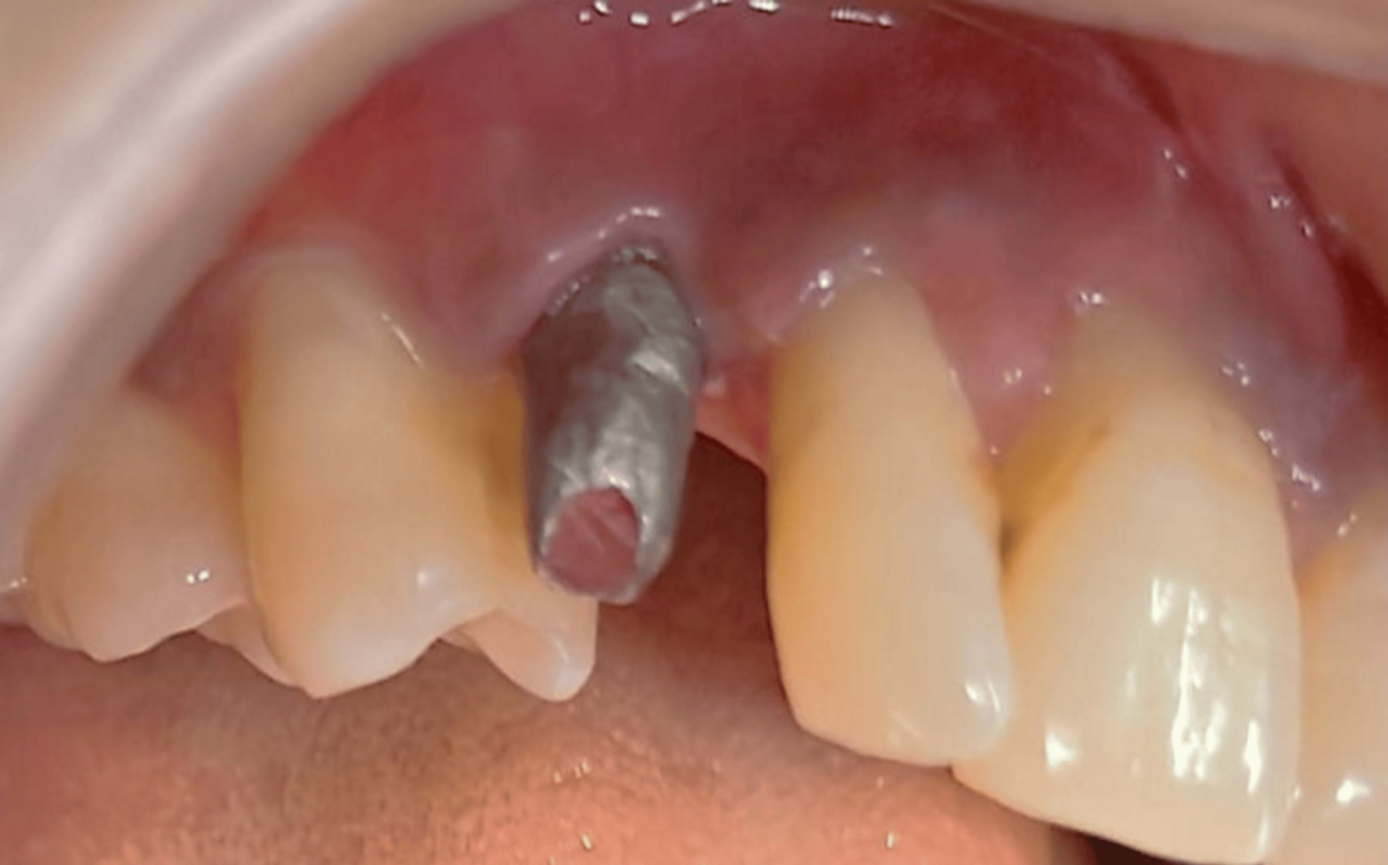
Placement of standard abutment after four months

**Figure 8:**
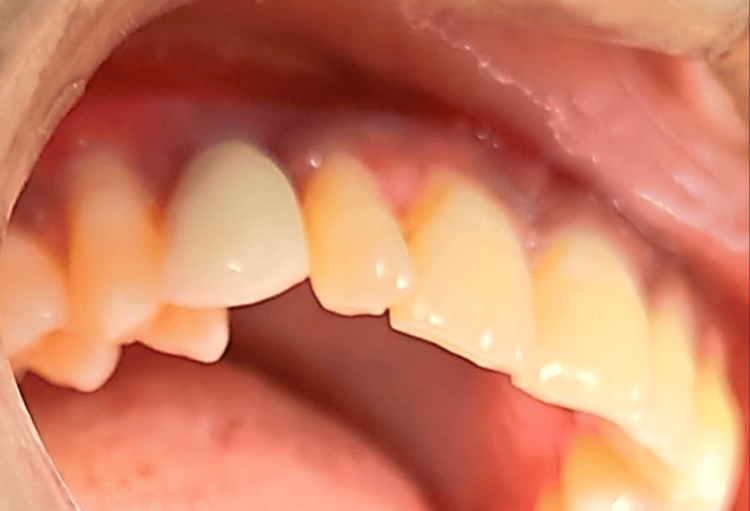
Cementation of porcelain fused to the metal crown

Prosthetic rehabilitation was scheduled to commence four months post-implant placement due to insufficient surrounding tissue. Hence, the implant was loaded after bone maturation and complete osseointegration. A standard abutment was placed after four months (Figure 7) and a final porcelain fused to metal (DMLS) crown was fabricated for canine and good emergence profile was achieved (Figure 8). After delivery of the definitive crown, the patient was included in a regular maintenance indicating that the implant was stable, and no peri-implant radiolucency was noted.

At the follow-up after two months, no bleeding on probing or pathological probing depths was observed, and only physiologic sulcus depths were seen. The peri-implant soft tissue level appeared to be stable, and the palatal gingival margin adapted to the tooth contours without signs of recession in all areas, including the canine extraction site. The interdental papillae were preserved, contributing to an optimal final aesthetic result. The patient would be recalled after one month for the next radiological examination. 

## Discussion

A labially impacted maxillary canine has a slight prevalence as compared to a palatally impacted canine and is typically attributed to the insufficient arch span. As a result, the canine is frequently positioned elevated within the alveolar bone and emerges through the alveolar mucosa [10]. A variety of techniques, like space recreation, orthodontic mechanical eruption, extraction, and interceptive approaches, were proposed to treat a labially or palatally impacted canine [11]. Regarding orthodontic therapy, there are three potential reasons for immobility during the intervention: (1) bone that is present surrounding the impacted crown; (2) improper orthodontic mechanics; and (3) ankylosis. If the forced eruption of the impacted tooth proves to be ineffective, alternative prosthetic solutions for rehabilitation should be evaluated, such as transalveolar extraction succeeded by immediate implant insertion or fixed or removable prostheses [12].

Fixed prosthodontic alternatives, such as cantilever or three-unit fixed dental prostheses, may be considered; nevertheless, these frequently necessitate the alteration of otherwise vital, non-carious, or previously rehabilitated teeth [13]. Also, both short-term and long-term survival rates for resin-bonded prostheses have been reported as uncertain [14]. Fournier et al. suggested that teeth with labial impaction positioned favorably in vertical orientation might be managed through surgical disclosure [15]. Immediate implant placement in the anterior region was introduced by Schulte and Heimke [16] in the late 1970s, as it can prevent buccal bone resorption, reduce the treatment duration, and enhance the aesthetic outcome of the final restoration [17]. They also reported comparable survival rates for immediate implants versus those placed in restored alveolar ridges. Immediate implant placement, if combined with ARP or guided regeneration procedures, gives satisfactory results and has a lot of advantages, such as reduced treatment time, number of dental appointments, and patient morbidity. In addition, it provides immediate aesthetics and is preferred by patients from a psychological point of view. Some authors have even claimed that the success rate of immediate implant placement is similar to that of conventional implantation [18]. However, it demands more precise surgical protocol and planning. A common aesthetic challenge with this approach is midbuccal gingival recession and the visibility of the metallic outline. To mitigate these concerns, many approaches were proposed like the use of tissue grafts, either alone or in combination with xenografts like bovine bone, as well as ceramic-derived alternatives. These methods function as barriers, preserving ridge width and volume, supporting soft tissue, and preventing its failure, thereby facilitating the reconstruction of the alveolar process, and improving aesthetic outcomes [19]. The same challenge was faced by clinicians in the present case as there was deficient bone buccally which was treated with complex bone grafting.

The present case report outlines a method for replacing an extracted labially impacted primary canine with an immediate implant. This procedure offers several advantages, such as preserving adjacent papillae, which contributes to an aesthetically pleasing result and minimizes marginal bone loss, thereby achieving an optimal relationship between the implant and the surrounding tissues. This case report aligns with existing research and literature supporting the placement of single implants in the anterior maxilla. However, it is important to account for potential limitations in surgical exposure, such as the requirement for bone grafts or membranes, unpredictable soft and hard tissue levels, and challenges in achieving adequate implant stability [20]. Further studies involving an expanded sample size are required to assess the short- and long-term results of this technique.

## Conclusions

The surgical elimination of an impacted maxillary canine, succeeded by its substitution with implant and grafting in conjunction with autogenous bone, xenograft, and bovine bone, represents an effective treatment strategy. This approach is particularly advantageous for adult individuals with impacted canines who are not candidates for orthodontic treatment, offering a novel solution for such cases. CBCT plays an important part in assessing the success of this treatment. Successful outcomes are contingent upon well-planned and meticulously executed surgical procedures, considering the position and progression of the impacted canine. Timely identification is crucial for promoting physiological canine emergence and optimizing implantation. Additional clinical trials are required to assess the long-term effectiveness of this technique.
